# Ethical Issues in Using Twitter for Public Health Surveillance and Research: Developing a Taxonomy of Ethical Concepts From the Research Literature

**DOI:** 10.2196/jmir.3617

**Published:** 2014-12-22

**Authors:** Mike Conway

**Affiliations:** ^1^University of California San DiegoDepartment of Family and Preventive MedicineLa Jolla, CAUnited States

**Keywords:** social media, twitter messaging, ethics

## Abstract

**Background:**

The rise of social media and microblogging platforms in recent years, in conjunction with the development of techniques for the processing and analysis of “big data”, has provided significant opportunities for public health surveillance using user-generated content. However, relatively little attention has been focused on developing ethically appropriate approaches to working with these new data sources.

**Objective:**

Based on a review of the literature, this study seeks to develop a taxonomy of public health surveillance-related ethical concepts that emerge when using Twitter data, with a view to: (1) explicitly identifying a set of potential ethical issues and concerns that may arise when researchers work with Twitter data, and (2) providing a starting point for the formation of a set of best practices for public health surveillance through the development of an empirically derived taxonomy of ethical concepts.

**Methods:**

We searched Medline, Compendex, PsycINFO, and the Philosopher’s Index using a set of keywords selected to identify Twitter-related research papers that reference ethical concepts. Our initial set of queries identified 342 references across the four bibliographic databases. We screened titles and abstracts of these references using our inclusion/exclusion criteria, eliminating duplicates and unavailable papers, until 49 references remained. We then read the full text of these 49 articles and discarded 36, resulting in a final inclusion set of 13 articles. Ethical concepts were then identified in each of these 13 articles. Finally, based on a close reading of the text, a taxonomy of ethical concepts was constructed based on ethical concepts discovered in the papers.

**Results:**

From these 13 articles, we iteratively generated a taxonomy of ethical concepts consisting of 10 top level categories: privacy, informed consent, ethical theory, institutional review board (IRB)/regulation, traditional research vs Twitter research, geographical information, researcher lurking, economic value of personal information, medical exceptionalism, and benefit of identifying socially harmful medical conditions.

**Conclusions:**

In summary, based on a review of the literature, we present a provisional taxonomy of public health surveillance-related ethical concepts that emerge when using Twitter data.

## Introduction

Since its inception in 2006, the microblog platform Twitter has become a key resource for understanding—and sometimes predicting—mass behavior, particularly in the area of marketing [1] and politics [2]. More recently, the public health community has recognized Twitter’s potential for public health surveillance [3,4] with applications including monitoring the prevalence of infectious diseases in the community [5,6], identifying early-stage disease outbreaks [7], detecting disease outbreaks in mass gatherings [8], and recognizing and understanding health behaviors, like temporal variability in problem drinking [9], and attitudes toward emerging tobacco products such as electronic cigarettes and hookah [10]. Despite the clear utility of using Twitter to augment current public health surveillance, there remains doubt among regulatory authorities, ethics committees, and individual researchers regarding ethically appropriate conduct in this kind of large-scale research, where a single researcher can automatically process hundreds of millions of public tweets. Adding to this difficulty is the fact that many Twitter researchers are based in university computer science and engineering departments, environments that often have not shared as long a tradition of ethical and regulatory oversight as health-related fields.

While there has been significant research effort in developing ethical guidelines for conducting Internet discussion forum-based research generally [11] and for developing ethical guidelines on appropriate use of social media for clinicians [12], there is little current work addressing ethical problems in large-scale automatic Twitter-based public health research. In this paper, we attempt to address this problem by systematically reviewing ethical content in Twitter-based public health surveillance papers with a view to: (1) explicitly identifying a set of potential ethical issues and concerns that may arise when researchers work with Twitter data, and (2) providing a starting point for the formation of a set of best practices for public health surveillance through the development of a taxonomy of ethical concepts derived from the research literature.

## Methods

**Figure 1:**
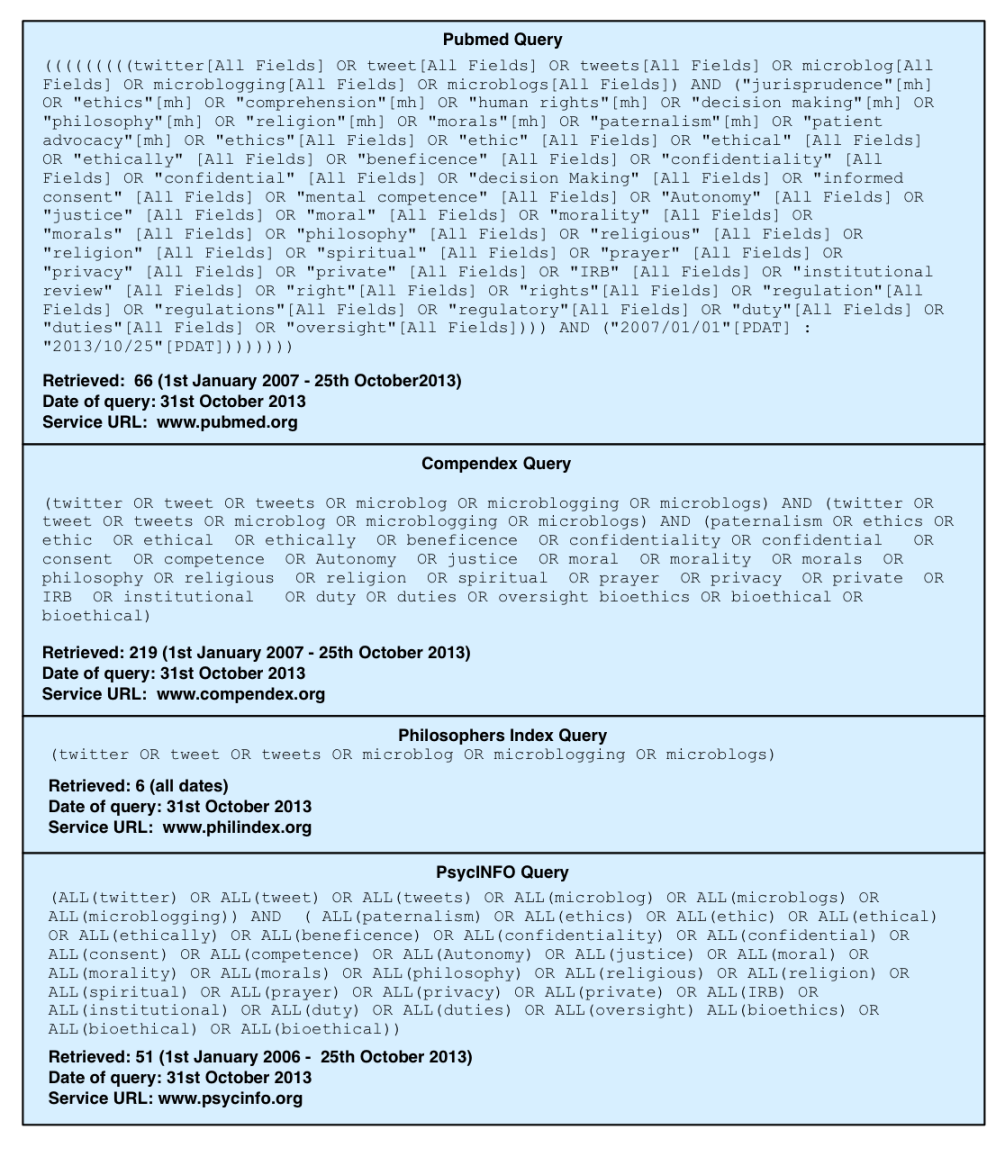
Literature search queries for PubMed, Compendex, Philosopher's Index, and PsycINFO.

In this review, we are focused on exploring ethical issues that have been identified in published Twitter-based public health surveillance research papers. Relevant research is dispersed across several broad research areas, including biomedicine (Medline), computer science and engineering (Compendex), philosophy (Philosopher’s Index), and psychology (PsycINFO). As we are primarily interested in ethical and regulatory issues and how they relate to public health surveillance, with the aid of a biomedical librarian, we designed a complex set of queries for each indexing service to identify those papers that included ethics-related terms in their titles or abstracts, such as “IRB” (institutional review board), “ethics”, and “privacy”. See Figure 1 for a complete list of keywords.

As the focus of this review is on ethical issues in large-scale automatic Twitter-based research for public health surveillance, we excluded work centered on non-microblog social media platforms (eg, Facebook). We also excluded work on policy and clinician/student professionalism (eg, proposed guidelines for governing clinician interaction with patients via Twitter), and research focused on non-health related topics (eg, marketing) with the exception of those articles concentrating on automatically identifying personality variables from Twitter feeds.

After searching the four databases with queries shown in Figure 1, we screened articles by titles and abstracts, discarding papers that were not available on an open-access basis or via the University of California library system. We began identifying ethical concepts by carefully reading two papers that, through our initial review, we identified as being especially rich in ethical content [13,14]. From these two initial papers, we highlighted sections of the text discussing ethical content and iteratively constructed an initial ethical taxonomy. We then carefully reviewed the remaining 11 papers, adding to and refining the taxonomy. Our methodology was inspired by, but is not identical to, that used by Strech et al [15] who used a rigorous grounded theory methodology to comprehensively investigate ethical issues in the dementia literature. Our aim in this short paper is limited to producing an outline of the ethical issues identified in the Twitter-based public health surveillance research literature.

## Results

### Overview

**Figure 2:**
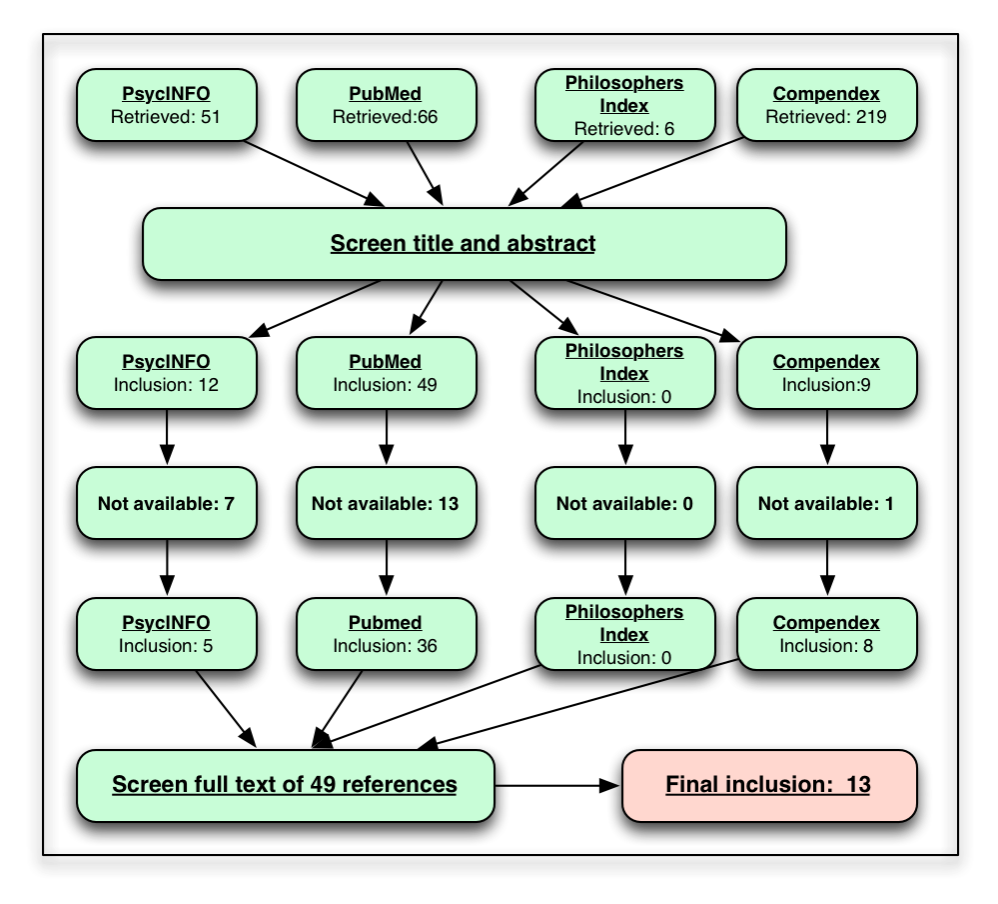
Inclusion/exclusion flowchart.

Our initial set of queries identified 342 references across the four databases (see Figure 2). After title and abstract screening, 49 references remained. After further full-text screening of these 49 references, 13 remained. Five of the papers were from biomedical journals [13,16-19] and six were from computer science and engineering conference proceedings [20-25]. One paper appeared in a journal dedicated to the social and cultural impact of technology [14]. Finally, one paper was published in the proceedings of a collaborative technology conference [26]. All articles were peer reviewed and written in English.

### Taxonomy

Our iterative, taxonomy construction process identified 10 broad ethical categories (eg, privacy, IRB/regulation). Six of these categories included several subcategories (eg, privacy has subcategories including the concept of privacy, IRB/regulation has subcategories including data protection legislation). The most prevalent and complex ethical category we found was privacy with 16 subcategories covering such important ethical concepts as unintended revelation of personal information and population level monitoring vs individual diagnosis (Textbox 1). Multimedia Appendix 1 shows example sentences and paragraphs for each ethical concept in the taxonomy.

**Ethical categories identified during the iterative, taxonomy construction process.**

1. Privacy
  a. Concept of privacy
  i. Public vs private - doubts about the location of Twitter data on the public/private spectrum [13,14,18,21,24]
  ii. Fluidity in the concept of privacy - rapidly changing concept of privacy [13,14,18,21,24]
  iii. Generational differences in the concept of privacy [13,18]
  iv. Panopticon effect - risk that public health monitoring will change user behavior on Twitter [24]
  b. Confidentiality
  i. Data linkage - risk of privacy loss due to linking data from different sources [13,21]
  ii. Confidentiality - appropriate storage of Twitter data by researchers [14]
  iii. Right to/desire for anonymity - research using Twitter challenges a participant’s right to (and desire for) anonymity [13,14,16]
  c. Stigmatized medical conditions - concerns about protecting the privacy of those with stigmatized medical conditions (eg, epilepsy, depression) [13,20]
  d. Twitter’s privacy policy - implications of Twitter’s privacy policy and how it is understood by users [14,18,19,21,22]
  e. Twitter is publicly accessible by default - emphasizes that Twitter is a broadcast medium. Unless a user changes privacy settings, tweets are public [14,19,21,22,24]
  f. Reliability of user provided personal details - reliability of information derived from Twitter when some users use false or whimsical personal details to maintain anonymity [19,21,24]
  g. Interpreting decontextualized Twitter data as fully representative of users who are in fact multifaceted - possibility that the user might be experimenting with self-presentation or exhibit a belief or behavior in their historical tweets that they no longer adhere to (eg, illegal drug use) [13,24]
  h. Unintended revelation of personal information - potential for a user to unintentionally provide insights into their mental health, health behaviors, etc, through information garnered from their tweets [20,22-24]
  i. Personal responsibility of Twitter users - emphasizes the responsibility users have for their posts [22,24]
  j. Twitter users have no expectation of privacy - emphasizes the researchers’ belief that Twitter users have no reasonable expectation of privacy [19,21]
  k. Identifying users’ mental health status or personality traits to:
  i. identify those in need of treatment [22,25]
  ii. job placement [20,22-24]
  iii. targeted marketing [21,23]
  iv. system interface design (eg, introverts prefer data presented in a certain way) [21,23]
  v. law enforcement (eg, identifying psychopaths) [22]
  l. Population level monitoring vs individual diagnosis - difference between using Twitter to identify broad, population level changes and diagnosing individuals [20]
  m. Potential for discrimination based on health status as garnered from social media [20]
  n. Danger of inaccurately labeling a user as suffering from a particular health problem [20,22]
  o. Traceability of Twitter data - risk that tweets can be traced back to the original tweeter if reproduced verbatim in research work, threatening anonymity [13,17]
  p. Intended audience for tweets - some Twitter users use Twitter as a communication tool for a small group of family and friends and do not expect their tweets to be widely read (ie, hidden in plain sight). Other Twitter users aim to broadcast to the world and gain the maximum number of followers [14,21,24]
2. Informed Consent
  a. Twitter users are oblivious or unwilling research participants [13]
  b. Informed consent is difficult (or impossible) to gain (or not required) for large-scale Twitter work [13,14,24]
3. Ethical Theory
  a. Difficulties in applying current ethical theories to mass Twitter research [13]
  b. Ethical theories:
  i. Deontology [13,26]
  ii. Utilitarianism [13,26]
  iii. Feminism [13]
  iv. Communitarianism [13]
  v. Application of the “golden rule” [13]
  vi. Agile/situational ethics [14]
  vii. Rawls’ theory of justice [26]
4. IRB/Regulation
  a. Citizens’ rights to communicate and share information [24,26]
  b. Researcher belief that regulatory oversight is not required when using Twitter data [19]
  c. Discussion of IRB/ethics committees, generally [14,18]
  d. Data protection legislation [14,18]
  e. Professional codes of conduct [14]
  f. Need for regulatory control, generally [18,20]
  g. Privacy regulation by country [14,24]
5. Traditional research vs Twitter research
  a. Apomediation - shifting from hierarchical models of research to a situation where the researcher is a potential participant [18]
  b. Scale of Twitter-based research - research norms that were developed for small-scale research do not scale to millions of Twitter users [14]
  c. Greater distance between researcher and participants - mass Twitter-based research increases the distance between researchers and participants [14]
  d. Ambiguous status of participants - the status of participants is more ambiguous than in traditional research (ie, are they consumers, participants, patients, service users, journalists, etc) [14]
  e. Increase in researcher power - in mass Twitter research, a single researcher has access to millions of Twitter users, hence increasing researcher power [14]
6. Geographical Information
  a. Tracking physical location - potential loss of privacy in tracking Twitter users’ physical locations [13,14,19,21]
  b. Appropriate geographical granularity – potential loss of privacy in reporting a Twitter user’s precise location, compared to their general location. For example, reporting that a Twitter user is *somewhere* in Los Angeles is very different to reporting their precise location in Los Angeles [14]
7. Researcher Lurking [13,14]
8. Economic Value of Personal Information [14]
9. Medical Exceptionalism - health-related matters are qualitatively different from other, non-medical areas and require special attention (and perhaps regulation) [25]
10. Benefit of Identifying Socially Harmful Medical Conditions [22]

### Normative Rules

In several of the papers under review, explicit normative rules were presented (or suggested) for conducting and reporting mass Twitter public health surveillance work (Textbox 2). Note that these rules are *discussed* but not necessarily *endorsed*.

**Explicit normative rules for mass Twitter public health surveillance work.**

1. When reporting research, avoid quoting directly from users’ Twitter streams. Paraphrases should be used [13].
2. Informed consent should be gained from participants [13].
3. Metadata (usernames, location data, etc) should not be disclosed [13].
4. Twitter-based work is human subjects research and requires that some form of appropriate IRB/ethical review take place [14].
5. Data collection should be logged and justified [14].
6. There should be parity between the researcher and participants (ie, the researcher’s tweets and their associated locations, if appropriate, should be public) [14].
7. Employment-related profiling for mental health conditions should only be performed in exceptional circumstances (eg, security critical roles) [24].
8. Consent should be gained from potential employees before employment-related profiling for mental health conditions is performed [24].

## Discussion

The main output of this research is a taxonomy of ethical concepts derived from close reading of the literature. The taxonomy will be used to help frame future interview-based qualitative research focused on Twitter users’ attitudes to the use of microblog data for public health surveillance and, in due course, inform the generation of a set of ethical guidelines for using Twitter for public health surveillance and research. We found that ethical theory was rarely mentioned in the reviewed papers and, when it was discussed, that discussion was typically brief. Only two papers [13,26] explicitly discuss the application of traditional ethical theories (eg, deontology, utilitarianism) to mass Twitter-based public health surveillance. As expected, the bulk of the ethical concepts we discovered were concerned with privacy [13,14,16-25], including frequent references to the fluid and changing nature of the concept of privacy [13,14,18,21,24], and more concretely, to Twitter’s privacy policies [14,18,19,21,22]. Discussion of IRBs and regulation (or the lack thereof) was also widespread in the literature [14,18-20,24,26]. Some topics were raised by a single research paper, for example, the idea that the ability to automatically process millions of tweets increases researcher power compared to traditional research methodologies [14], and the idea that the benefits of using Twitter for public health purposes are so great that they mitigate any ethical doubts that would apply to other, non-health-related uses of Twitter data (eg, for commercial gain) [25].

Although inspired by the ethics-oriented qualitative literature review methodology proposed by Strech [15], the approach taken in this review is substantially different, in particular the use of a single reviewer (author MC) rather than a group of reviewers, and the use of *close reading* in place of a theoretically grounded qualitative methodology. A further characteristic of this review is that our search strategy was confined to papers indexed in PubMed, Compendex, PsycINFO, and the Philosopher’s Index. Papers that were not available via the University of California San Diego library system or on an open-access basis were excluded. It is likely that we “missed” relevant papers in business disciplines or in those computer science and engineering conferences and journals not indexed by Compendex. However, our purpose in this review was the identification of a broad taxonomy of ethical concepts relevant to Twitter-based public health research using a systematic, reproducible methodology and thus comprehensiveness, while desirable, is not a necessity.

In conclusion, this short paper provides a taxonomy of ethical concepts derived from the research literature. Future work will involve interview-based qualitative research exploring Twitter users’ attitudes toward the mining of their data for public health purposes, and ultimately the formation of best practice guidelines for public health surveillance using Twitter data.

## Multimedia Appendix 1

Taxonomy.

## Abbreviations

IRB: institutional review board
