# Supplementary material for: Ethical Issues in Using Twitter for Public Health Surveillance and Research: Developing a Taxonomy of Ethical Concepts From the Research Literature
Source: J Med Internet Res. 2014 Dec 22;16(12):e290. doi: 10.2196/jmir.3617 (PMC4285736; doi:10.2196/jmir.3617)
Supplement: Supplementary file 1 [file jmir_v16i12e290_app1.PDF]

Appendix

This document details the range of ethical issues found in Twitter health related papers with illustrative quotations. See paper *Ethical Issues in using Twitter for Public Health Surveillance and Research: Developing a Taxonomy of Ethical Concepts from the Research Literature*.

Contents

1 Privacy **3**

1.1 Concept of privacy . . . 3

1.1.1 public vs. private . . . 3

1.1.2 fluidity in the concept of privacy . . . 3

1.1.3 generational differences in the concept of privacy . . . 3

1.1.4 panopticon effect . . . 3

1.2 Confidentiality . . . 3

1.2.1 data linkage . . . 3

1.2.2 confidentiality . . . 4

1.2.3 right to/desire for anonymity . . . 4

1.3 Stigmatized medical conditions . . . 4

1.4 Twitter's privacy policy . . . 4

1.5 Twitter is publicly accessible by default . . . 4

1.6 Reliability of user provided personal details . . . 5

1.7 Interpreting decontextualized Twitter data as fully representative of users who are in fact multifaceted . . . 5

1.8 Unintended revelation of personal information . . . 5

1.9 Personal responsibility of Twitter users . . . 5

1.10 Twitter users have no expectation of privacy . . . 5

1.11 Identifying users mental health status or personality traits to: . . . 6

1.11.1 identify those in need of treatment . . . 6

1.11.2 job placement . . . 6

1.11.3 targeted marketing . . . 6

1.11.4 system interface design . . . 6

1.11.5 law enforcement . . . 6

1.12 Population level monitoring vs. individual diagnosis . . . 6

1.13 Potential for discrimination based on health status as garnered from social media . . . 7

1.14 Danger of inaccurately labeling a user as suffering from a particular health problem . . . 7

1.15 Traceability of Twitter data . . . 7

1.16 Intended audience for tweets . . . 7

2 Informed Consent **7**

2.1 Twitter users are oblivious or unwilling research participants . . . 7

2.2 Informed consent is difficult (or impossible) to gain (or not required) for large scale Twitter work . . . 8

3 Ethical Theory **8**

3.1 Difficulties in applying current ethical theories to mass Twitter research 8
3.2 Ethical theories: 8
3.2.1 Deontology 8
3.2.2 Utilitarianism 8
3.2.3 Feminism 9
3.2.4 Communitarianism 9
3.2.5 Application of the "golden rule" 9
3.2.6 Agile/situational ethics 9
3.2.7 Rawl's theory of justice 9

4 IRB/Regulation **10**
4.1 Citizens' rights to communicate and share information 10
4.2 Researcher belief that regulatory oversight is not required when using Twitter data 10
4.3 Discussion of IRB/Ethics committees, generally 10
4.4 Data protection legislation 10
4.5 Professional codes of conducts 10
4.6 Need for regulatory control, generally 10
4.7 Privacy regulation by country 11

5 Traditional Research vs Twitter Research **11**
5.1 Apomediation 11
5.2 Scale of Twitter-based research 11
5.3 Greater distance between researcher and participants 11
5.4 Ambiguous status of participants 11
5.5 Increase in researcher power 12

6 Geographical Information **12**
6.1 Tracking physical location 12
6.2 Appropriate geographical granularity 12

7 Researcher Lurking **12**

8 Economic Value of Personal Information **13**

9 Medical Exceptionalism **13**

10 Benefit of Identifying Socially Harmful Medical Conditions **13**

1 Privacy

1.1 Concept of privacy

1.1.1 public vs. private

References: [1, 2, 3, 4, 5]

Illustrative quotation: "Such findings indicate that the once clear lines between the private and the public, as well as the employee's personal and professional life are gradually blurring..." [2]

1.1.2 fluidity in the concept of privacy

References: [1, 2, 3, 4, 5]

Illustrative quotation: "I argue that, although privacy will always remain a valid concern, the apomediation of health information may herald (or have been caused by) a shift in moral norms in which privacy no longer means what it once did" [5]

1.1.3 generational differences in the concept of privacy

References: [4, 5]

Illustrative quotation: "This is an area that clearly needs much more research and it cannot be assumed that those who are willing to place so much of their private lives on the internet will have the same concerns as previous generations who might guard their privacy much more jealously." [4]

1.1.4 panopticon effect

References: [2]

Illustrative quotation: "The panoptic effect of being constantly monitored even concerning activities that fall out of the workplace frame has negative impacts on the employer-employee relationship that should be based on mutual trust and confidence." [2]

1.2 Confidentiality

1.2.1 data linkage

References: [1, 4]

Illustrative quotation: "Advances in data linkage, in which social media messages are combined with information on the location of the originator and the characteristics of the environment where they are located offer many additional possibilities." [4]

1.2.2 confidentiality

References: [3]

Illustrative quotation: "Primary among the procedural mechanisms to protect individuals involved in research are privacy, confidentiality, anonymity and informed consent." [3]

1.2.3 right to/desire for anonymity

References: [6, 3, 4]

Illustrative quotation: "Anonymity is a fundamental right of subjects of research. It underpins the potentially fragile trust between the subject and the researcher and is integral to consent and provision of information as well as being a manifestation of the respect in which the researcher holds the subject in front of the computer screen. The use of online material as data intensifies the challenges that exist in traditional settings. Thus, a quotation from an interview need not identify the subject but, if from a Tweet, it can be traced back to the subject in seconds using Google" [4]

1.3 Stigmatized medical conditions

References: [7, 4]

Illustrative quotation: "The personality construct of psychopathy has begun to be studied in combination with Machiavellianism and narcissism. The three constructs are overlapping, but distinct, and have been named the Dark Triad of personality because they all focus, to varying degrees on social malevolence, self-promotion, emotional coldness, duplicity and aggressiveness." [7]

1.4 Twitter's privacy policy

References: [8, 1, 9, 3, 5]

Illustrative quotation: "By design, this network does not focus on complex levels of privacy and sharing with different subsets of users: with only two privacy settings (all posts shown to a limited set of users or all posts public), most users (93.9%) opt to publish everything to everyone." [9]

1.5 Twitter is publicly accessible by default

References:[8, 1, 9, 2, 3]

Illustrative quotation: "By design, this network does not focus on complex levels of privacy and sharing with different subsets of users: with only two privacy settings (all posts shown to a limited set of users or all posts public), most users (93.9%) opt to publish everything to everyone." [9]

1.6 Reliability of user provided personal details

References:[8, 1, 2]

Illustrative quotation: "Location is an optional text field in which users can enter anything they want. Many users provide their geographical position, such as a city and state/country, but many opt to specify something humorous (e.g., somewhere in my imagination :) or a cube world in Minecraft), sarcastic (e.g., in yhur [bleep!!!] face or Here...obvious!), or just leave the field blank." [8]

1.7 Interpreting decontextualized Twitter data as fully representative of users who are in fact multifaceted

References: [2, 4]

Illustrative quotation: "De-contextualization is an inherent characteristic of social media that pertains to over-simplification of social relations and the wide dissemination of information." [2]

1.8 Unintended revelation of personal information

References: [7, 9, 10, 2]

Illustrative quotation: "Methods (such the proposed in this paper) allows employers to collect and aggregate information, which reflects behavior of the user and her interaction with other users, in order to produce relevant patterns/profiles and anticipate future behaviors and threats." [2]

1.9 Personal responsibility of Twitter users

References: [9, 2]

Illustrative quotation: "Because of this open sharing model, some feel that Twitter has few privacy implications: users know that everything is public, and if they still choose to post embarrassing content, this in not a technological problem, but a social one" [9]

1.10 Twitter users have no expectation of privacy

References: [8, 1]

Illustrative quotation: "Because of the public nature of the tweets, users do not have any expectation of privacy, so researchers may openly observe the content". [8]

1.11 Identifying users mental health status or personality traits to:

1.11.1 identify those in need of treatment

References: [11, 9]

Illustrative quotation: "Although the results are not sufficiently accurate to perform diagnosis or support direct action against those predicted as problematic, they do enable flagging of potentially risky individuals." [9]

1.11.2 job placement

References: [7, 9, 10, 2]

Illustrative quotation: "Naturally, there are circumstances where it would be advantageous to identify those who score highly for psychopathy, including employment..." [9]

1.11.3 targeted marketing

References: [1, 10]

Illustrative quotation: "With the ability to guess a users personality traits, many opportunities are opened for personalizing interfaces and information. We discussed some of these opportunities for marketing and interface design above." [10]

1.11.4 system interface design

References: [1, 10]

Illustrative quotation: "With the ability to guess a users personality traits, many opportunities are opened for personalizing interfaces and information. We discussed some of these opportunities for marketing and interface design above." [10]

1.11.5 law enforcement

References: [9]

Illustrative quotation: "Overall, we show that data mining can be a valuable tool for law enforcement and others interested in identifying abnormal psychiatric states from Twitter data." [9]

1.12 Population level monitoring vs. individual diagnosis

References: [7]

Illustrative quotation: "While predictive models may be unsuitable for predicting an individuals personality, they may still be of practical importance when models are applied to large groups of people, such as gaining the ability to see whether anti-social traits are increasing or decreasing over a population." [7]

1.13 Potential for discrimination based on health status as garnered from social media

References: [7]

Illustrative quotation: "Given the potential real-world uses of this information, e.g. pre-employment screening, and despite discrimination and invasion of privacy issues, this inconsistency should be addressed." [7]

1.14 Danger of inaccurately labeling a user as suffering from a particular health problem

References: [7, 9]

Illustrative quotation: "Since social media personality prediction could be used to label an individual, it is important to ensure the correct evaluation metrics are selected in research studies." [7]

1.15 Traceability of Twitter data

References: [12, 4]

Illustrative quotation: "The study had ethical approval and steps were taken to ensure anonymity. However the college was easily identified, making it possible to trace individuals. Even when such data are anonymised, it can be relatively easy to combine variables in ways that identify individuals." [4]

1.16 Intended audience for tweets

References: [1, 2, 3]

Illustrative quotation: "However, while this setting to share tweets among a select group creates a sense of closed community and might lead one to believe that the information or data sent using this platform can only be read and accessed by the circle of followers (e.g. friends), this is patently not the case . . . Every Twitter message sent is public, unless specifically sent as a private message." [3]

2 Informed Consent

2.1 Twitter users are oblivious or unwilling research participants

References: [4]

Illustrative quotation: “While the researcher might post information on his or her public profiles to be shared by friends or peers, this does not mean that they have consented for this information to be collated, analysed, and published, in effect turning them into research subjects” [4]

2.2 Informed consent is difficult (or impossible) to gain (or not required) for large scale Twitter work

References: [2, 3, 4]

Illustrative quotation: “Now it is possible to consider research conducted not with 1,000 or 10,000 participants but with 100,000 or one million individual users. The relations between researchers and participants are now one or a few to the multitudes. With large data sets, the individual disappears in the mass, but the issue and potential risk remain on the scale of the individual. Moreover, IRB protocol measures for informed consent are simply not viable at this scale.” [3]

3 Ethical Theory

3.1 Difficulties in applying current ethical theories to mass Twitter research

References: [4]

Illustrative quotation: “However, the speed with which these new sources of data [i.e. social media] have emerged, as well as the increasingly imaginative ways that researchers are using them, risked running ahead of the development of an appropriate ethical framework for their use.” [4]

3.2 Ethical theories:

3.2.1 Deontology

References: [13, 4]

Illustrative quotation: “Ethicists have recognized that they face a challenge in determining how to transfer traditional deontological principles into the world of social media, addressing the duties and obligations of the researcher, as well as how to deal with concepts such as utilitarianism, feminism, and communitarianism.” [4]

3.2.2 Utilitarianism

References: [13, 4]

Illustrative quotation: “Ethical considerations of citizen participation over Twitter in response to violent crises will be discussed based on theories drawn from Rawl’s theory of justice, Kant’s categorical imperative, and Mill’s view on utilitarianism” [13]

3.2.3 Feminism

References: [4]

Illustrative quotation: "Ethicists have recognized that they face a challenge in determining how to transfer traditional deontological principles into the world of social media, addressing the duties and obligations of the researcher, as well as how to deal with concepts such as utilitarianism, feminism, and communitarianism." [4]

3.2.4 Communitarianism

References: [4]

Illustrative quotation: "Ethicists have recognized that they face a challenge in determining how to transfer traditional deontological principles into the world of social media, addressing the duties and obligations of the researcher, as well as how to deal with concepts such as utilitarianism, feminism, and communitarianism." [4]

3.2.5 Application of the "golden rule"

Reference: [4]

Illustrative quotation: "One approach is to apply the ethic of reciprocity, or Golden Rule, whereby the researcher asks how they would feel if the roles were reversed." [4]

3.2.6 Agile/situational ethics

References: [3]

Illustrative quotation: "We conclude by translating principles of agile software development into agile ethics. Rather than a defined set of codes or bureaucratic machinery to ensure ethical conduct from the top-down, this mode of engagement has more in common with what has been described as an in situ creative and collaborative ethical practice that works bottom-up." [3]

3.2.7 Rawl's theory of justice

References: [13]

Illustrative quotation: "Ethical considerations of citizen participation over Twitter in response to violent crises will be discussed based on theories drawn from Rawl's theory of justice, Kant's categorical imperative, and Mill's view on utilitarianism" [13]

4 IRB/Regulation

4.1 Citizens' rights to communicate and share information

References: [13, 2]

Illustrative quotation: "Discussions will also describe citizens rights in communicating and sharing information as protected by law." [13]

4.2 Researcher belief that regulatory oversight is not required when using Twitter data

References: [8]

Illustrative quotation: "Because of the public nature of the tweets, users do not have any expectation of privacy, so researchers may openly observe the content" [8]

4.3 Discussion of IRB/Ethics committees, generally

References[3, 5]

Illustrative quotation: "This is because, at root, the gaps in IRB protocols when applied to such massified research cannot be filled." [3]

4.4 Data protection legislation

References:[3, 5]

Illustrative quotation: "Informed by subsequent national and international data-protection legislation, the implementation and enforcement of professional codes of conduct mean that researchers must negotiate a series of overlapping legal, institutional and professional protocols." [3]

4.5 Professional codes of conducts

References: [3]

Illustrative quotation: "Informed by subsequent national and international data-protection legislation, the implementation and enforcement of professional codes of conduct mean that researchers must negotiate a series of overlapping legal, institutional and professional protocols." [3]

4.6 Need for regulatory control, generally

References:[7, 5]

Illustrative quotation: "This points to critical questions around the possible need for regulatory controls and/or raising awareness amongst users in order to prevent the misuse of information derived from Twitter and other online social network activity." [7]

4.7 Privacy regulation by country

References: [2, 3]

Illustrative quotation: "Regarding privacy as a purely bargainable and alienable right ignores the dignity element, inherent in the notion of privacy. The European approach seems diametrically opposite in many respects: Privacy is not conceived as a right to seclusion and intimacy but as a phenomenon, a protectable situation that regards the relationships between a person and its environment/other persons." [2]

5 Traditional Research vs Twitter Research

5.1 Apomediation

References: [5]

Illustrative quotation: "In the new apomediated world, it is increasingly difficult to tell the difference between the researcher and the subject, begging the question: if regulations are there to protect subjects from researchers, what are regulations for when subject and research seem to be one and the same?" [5]

5.2 Scale of Twitter-based research

References: [3]

Illustrative quotation: "Now it is possible to consider research conducted not with 1,000 or 10,000 participants but with 100,000 or one million individual users." [3]

5.3 Greater distance between researcher and participants

References: [3]

Illustrative quotation: "The relations between researchers and participants are now one or a few to the multitudes. With large data sets, the individual disappears in the mass, but the issue and potential risk remain on the scale of the individual." [3]

5.4 Ambiguous status of participants

References: [3]

Illustrative quotation: "Further implications to consider have to do with internal relations that comprise the identities of the participant, the researcher and that of the data. What is the status of the participant?" [3]

5.5 Increase in researcher power

References: [3]

Illustrative quotation: "As we have seen with Twitter's API, a researcher can conveniently collect data from his/her computer without the knowledge of the data producer or the participant. Where IRB protocols could aid in maintaining a direct and formally recognized agreement between the two parties, now there is often no interaction between the two parties involved. We might say this is a change in the external relations of this type of research. These relations are now much more anonymous. In terms of accountability, the issue of (researcher) anonymity now becomes a problem rather than being a solution for minimizing risk to the individual in conventional research." [3]

6 Geographical Information

6.1 Tracking physical location

References:[8, 1, 3, 4]

Illustrative quotation: "Tracking of online activities, in terms of content, activity, time and physical location, is increasingly possible. This is especially so with Twitter and similar location-based media platforms." [3]

6.2 Appropriate geographical granularity

References: [3]

Illustrative quotation: "While the NCL maps are based on individual Twitter messages, the data have been aggregated and the resulting visualization is a density surface generated from the tweets. Consequently, the individual tweet no longer features in the output. Even if, for example, we show the location of an individual message as in the visualizations from a map of London generated with Twitter usage (Figure 4), the resolution of the clip in pixels is so low that it becomes nearly impossible to determine a precise location." [3]

7 Researcher Lurking

References: [3, 4]

Illustrative quotation: "The nature of social media means that researchers can now lurk in wait for what are, in essence, ready-made data sets." [4]

8 Economic Value of Personal Information

Reference: [3]

Illustrative quotation: "As described above using the example of Twitter, the issue of privacy is not clear cut. This is because within the information economy, privacy is perceived by users in one manner, yet is handled by the online service provider in a very different manner." [3]

9 Medical Exceptionalism

References: [11]

Illustrative quotation: "Although the methods may be based in a similar mathematics of statistics and large-scale data analysis about people and their online activities, predictions about future changes in psychological wellbeing may be viewed by many as qualitatively different. People may be uncomfortable with the possibility that third parties might have the ability to predict future psychological states, especially when relatively accurate predictions can be made about future illness and disability. We believe it is important to bring the possibilities to the fore, so as to leverage the benefits of these methods and ideas to enhance the quality of life for people, as well as to stimulate discussion and awareness of potential concerns that need to be addressed at the individual and societal levels." [11]

10 Benefit of Identifying Socially Harmful Medical Conditions

References: [9]

Illustrative quotation: "Discovering psychopathic individuals has implications beyond simply determining gender or political leanings: such individuals may pose a danger towards others, or at least might be best approached with caution." [9]

References

[1] Quercia D, Kosinski M, Stillwell D, Crowcroft J. Our Twitter profiles, our selves: Predicting personality with Twitter. Proceedings of the Institute of Electrical and Electronics Engineers International Conference on Privacy, Security, Risk and Trust, PASSAT 2011. 2011;p. 180–185. DOI:10.1109/PASSAT/SocialCom.2011.26.

[2] Kandias M, Galbogini K, Mitrou L, Gritzalis D. Insiders trapped in the mirror reveal themselves in social media. Proceedings of the 7th International Conference on Network and System Security. 2013;7873 LNCS:220–235. DOI:10.1007/978-3-642-38631-2_17.

[3] Neuhaus F, Webmoor T. Agile ethics for massified research and visualization. Information, Communication & Society. 2012;15(1):43–65. DOI:10.1080/1369118X.2011.616519.

[4] McKee R. Ethical issues in using social media for health and health care research. Health Policy. 2013 May;110(2-3):298–301. PMID:23477806.

[5] O'Connor D. The apomediated world: regulating research when social media has changed research. J Law Med Ethics. 2013;41(2):470–83. DOI:10.1111/jlme.12056.

[6] Sugawara Y, Narimatsu H, Hozawa A, Shao L, Otani K, Fukao A. Cancer patients on Twitter: a novel patient community on social media. BMC Res Notes. 2012;5:699. PMID: 23270426.

[7] Sumner C, Byers A, Boochever R, Park GJ. Predicting dark triad personality traits from Twitter usage and a linguistic analysis of tweets. Proceedings of the 11th IEEE International Conference on Machine Learning and Applications. 2012;2:386–393. DOI:10.1109/ICMLA.2012.218.

[8] Burton SH, Tanner KW, Giraud-Carrier CG, West JH, Barnes MD. "Right time, right place" health communication on Twitter: value and accuracy of location information. J Med Internet Res. 2012;14(6):e156. PMID:23154246.

[9] Wald R, Khoshgoftaar TM, Napolitano A, Sumner C. Using Twitter content to predict psychopathy. Proceedings of the 11th Institute of Electrical and Electronics Engineers International Conference on Machine Learning and Applications. 2012;2:394–401. DOI:10.1109/ICMLA.2012.228.

[10] Golbeck J, Robles C, Edmondson M, Turner K. Predicting personality from Twitter. Proceedings of the Institute of Electrical and Electronics Engineers International Conference on Privacy, Security, Risk and Trust. 2011;p. 149–156. DOI:10.1109/PASSAT/SocialCom.2011.33.

[11] De Choudhury M, Counts S, Horvitz E. Predicting postpartum changes in emotion and behavior via social media. Proceedings of the 31st Annual CHI Conference on Human Factors in Computing Systems: Changing Perspectives. 2013;p. 3267–3276. DOI:10.1145/2470654.2466447.

[12] Heaivilin N, Gerbert B, Page JE, Gibbs JL. Public health surveillance of dental pain via Twitter. J Dent Res. 2011 Sep;90(9):1047–51. PMID: 21768306.

[13] Heverin T. Ethical concerns of Twitter use for collective crisis response. Proceedings of the 12th International Conference on Collaboration Technologies and Systems. 2011;p. 625–626. DOI:10.1109/CTS.2011.5928746.
